# Comprehensive Biomechanism of Impact Resistance in the Cat's Paw Pad

**DOI:** 10.1155/2019/2183712

**Published:** 2019-07-31

**Authors:** Xueqing Wu, Baoqing Pei, Yuyang Pei, Yan Hao, Kaiyuan Zhou, Wei Wang

**Affiliations:** ^1^School of Biological Science and Medical Engineering, Beihang University, Beijing, 100083, China; ^2^Beijing Advanced Innovation Centre for Biomedical Engineering, Beihang University, Beijing, 100083, China; ^3^School of Public Health, Nanjing Medical University, Nanjing, 211166, China

## Abstract

Cats are able to jump from a high-rise without any sign of injury, which is attributed in large part to their impact-resistant paw pads. The biomechanical study of paw pads may therefore contribute to improving the impact resistance of specific biomimetic materials. The present study is aimed at investigating the mechanics of the paw pads, revealing their impact-resistant biomechanism from macro- and microscopic perspectives. Histological and micro-CT scanning methods were exploited to analyze the microstructure of the pads, and mechanical testing was conducted to observe the macroscopic mechanical properties at different loading frequencies. Numerical micromodels of the ellipsoidal and cylindrical adipose compartments were developed to evaluate the mechanical functionality as compressive actions. The results show that the stiffness of the pad increases roughly in proportion to strain and mechanical properties are almost impervious to strain rate. Furthermore, the adipose compartment, which comprises adipose tissue enclosed within collagen septa, in the subcutaneous tissue presents an ellipsoid-like structure, with a decreasing area from the middle to the two ends. Additionally, the finite element results show that the ellipsoidal structure has larger displacement in the early stage of impact, which can absorb more energy and prevent instability at touchdown, while the cylindrical structure is more resistant to deformation. Moreover, the Von Mises of the ellipsoidal compartment decrease gradually from both ends to the middle, making it change to a cylindrical shape, and this may be the reason why the macroscopic stiffness increases with increasing time after contact. This preliminary investigation represents the basis for biomechanical interpretation and can accordingly provide new inspirations of shock-absorbing composite materials in engineering.

## 1. Introduction

Cats are generally acknowledged to have excellent athletic ability, especially in jumping, achieved through natural selection. When striking the ground, they can land smoothly, without any injury, though they are subjected to large impact forces, as high as several times their body weight [1, 2]. It is believed that the paw pads play a protective and load-bearing role during landing since they are the only body parts that touch the ground. Indeed, most sporty members of the Felidae family (including cats, tigers, leopards and so on) are extant representatives of the padded foot. The cat paw pad consists of digital and metacarpal pads, which are usually located beneath distal interphalangeal joints and metacarpophalangeal joints, respectively [3]. It is logical to argue that cats mainly rely on the metacarpal pads to absorb impact energy because they have relatively long carpals and tarsals as well as large metacarpal pads.

In the past few decades, a large number of studies have been done on the mechanical properties of human heel pads, revealing the nonlinear, viscoelastic mechanical behavior [4–7]. Moreover, the heel pad comprises adipose tissue enclosed within collagen septa, resulting in many small compartments, which are considered to be many small hydrostatic systems [8, 9]. Besides, the mechanical properties of heel pads have been found to be almost impervious to strain rate and temperature, suggesting that there is no bulk fluid flow between the small compartments [10]. Therefore, the compartment can be regarded as filled with fluid and the total volume remains unchanged, but deformation occurs during load-bearing. Additionally, different numerical models were developed accounting for the variation of adipose chambers dimensions, connective septae wall thickness, and fibre orientation, evaluating the overall mechanical response of calcaneal fat pad region under unconfined compression tests [11]. Furthermore, in a comparison of outcomes from numerical investigations on the infrapatellar fat pad (IFP), knee (KSF), and abdominal (ASF) subcutaneous fat tissues, the authors pointed out a similar mechanical behavior of IFP and KSF, while mechanical properties of ASF confirm a different response depending on both material and geometrical conformation characteristics [12].

Nevertheless, there have been few systematic studies on the biomechanical behaviour of cat paw pads. Alexander et al. [13] conducted the numerical simulation and vitro dynamic compression tests on the paw pads of some mammals, and the results show that the paw pads should have a variable mechanical property, so as to prevent the excessive ground peak reaction force and enhance stability and robustness under vibration. Meanwhile, a study on functional morphology and biomechanics of mammalian footpads indicates that the footpads should have a certain flexibility, stiffness, and damping, in order to absorb energy, transfer the ground reaction force, and maintain stability, respectively [14]. Additionally, the paw pad structures in the Clouded Leopard and Domestic Cat have been studied [15], showing that, covered by a stratified squamous cornified epithelium, the pads have a supple deposit of subepidermal fat that is partitioned by collagen fibers and extensively anchored to the muscle tendon sheaths.

As stated above, there is currently sufficient evidence to elucidate the important role of cat paw pads in energy absorption during impact. However, to our knowledge, there is a lack of comprehensive and comparative studies on cats' paw pads and no quantitative investigation of the micromechanics of paw pads has been published. It is known that the paw pads are composite materials, and their observed mechanical properties are achieved by the interaction of the various components. Studies of micromechanics (the ways in which the components interact), based on finite element analysis, can thus provide comprehensive insight into the internal buffering mechanism of cat paw pad during impact. In previous studies, the microstructure of the paw pads was concentrated in the two-dimensional morphology, but the minimum three-dimensional structural unit of load-bearing was not proposed. As a result, it is not well established that this information is sufficient to provide representative interpretation of impact resistance in the cat's paw pad.

The objective of this paper is therefore to study the comprehensive biomechanism of impact resistance in the cat's paw pad. In this study, a mechanical testing system was used to observe the macroscopic mechanical properties at different vibration frequencies, and the microstructure were investigated using section staining technique and micro-CT scanning. Additionally, finite element models of ellipsoidal and cylindrical (for control) adipose compartments, which are considered to be small hydrostatic systems, were established to study internal buffering mechanism of cat paw pad, making it possible to visualize the events that occur during impact. The results of this study will help to interpret and understand the impact resistance biomechanism of cat paw pads. A more practical motivation for this study is to provide useful information for the future development of impact resistant biomaterials.

## 2. Materials and Methods

### 2.1. Ethical Statement

A total of five domestic cats (2.45 ± 0.29 years of age, 3.6 ± 0.35 kg) that had died of heart disease were included in the study. After their death, the metacarpal pads of the forelimbs were removed and preserved. All experimental procedures were approved by the Science and Ethics Committee of Beihang University.

### 2.2. Histological Examination

The metacarpal pads of the left forelimbs of two cats were fixed in 10% formalin solution. Ten-micrometer-thick sections in the sagittal planes were obtained from the paraffin-embedded samples and stained with hematoxylin-eosin (HE), Verhoeff-van Gieson for elastic fibers, and Sirius red for histological examination. In particular, the subtypes of collagen were studied in the Sirius red staining sections using a polarized light microscope and recorded with a high-resolution digital camera able to differentiate type I collagen fibers, which appear orange to red, from the thinner type III collagen fibers, which appear yellow to green [16]. All images acquired from stained sections were then measured using ImageJ software, to estimate the amount of elastic fibers, type I and type III collagen fibers [17].

### 2.3. Micro-CT Scanning

In order to accurately investigate the two-dimensional morphology and three-dimensional structure, the metacarpal pads of the left forelimbs of the remaining three cats were scanned by micro-CT (Skyscan1272, Skyscan, Belgium) at a spatial resolution of 8 *μ*m. Finally, the scanned Dicom images were compared with the previous histological images and then imported to Mimics 17.0 for threshold segmentation and 3D reconstruction.

### 2.4. Mechanical Testing

**Figure 1:**
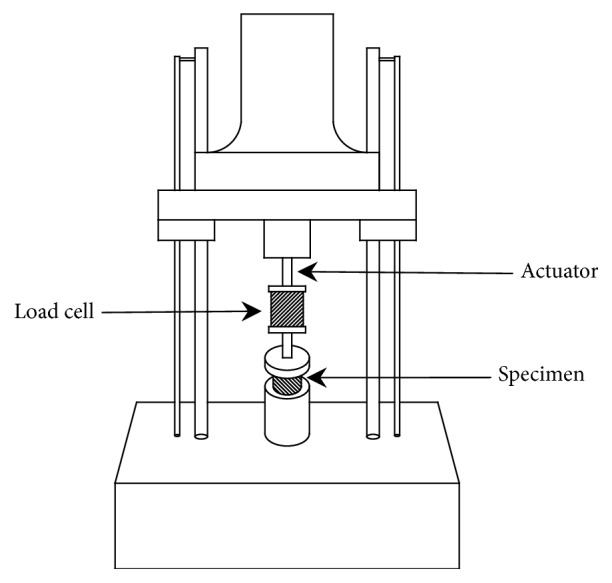
A diagram showing how the paw pads were mounted in the dynamic testing machine.

The metacarpal pads of the right forelimbs were slowly thawed to room temperature and cut into cylindrical samples with a diameter of 7 mm by a corneal trephine. Then the five pad samples with heights of 5.2 mm, 5.4 mm, 5.5 mm, 5.7 mm and 5.9 mm, were subjected to dynamic compressive tests, using Instron E10000 testing machine.  Figure 1 shows how they were mounted in the machine. They were sandwiched between steel or aluminium plates which were usually constrained to be parallel (Figure 1).

Before the mechanical testing, we read some typical articles on the mechanical behaviour of human and mammal heel (paw) pads [13, 18], finding that Instron testing machines were adopted to conduct the compression tests at 0.11Hz, 1.1Hz, and 11Hz. In order to ensure the reliability and comparability of our results, we also used the Instron testing machine to perform the tests at the three loading frequencies. Additionally, the three loading frequencies are common in different sports as walking, running, and jumping, respectively. The selection can make the mechanical analysis of the pad more comprehensive. Therefore, the actuator was made to move up and down sinusoidally under position control, where the loading frequencies were 0.11 Hz, 1.1 Hz, and 11 Hz, respectively, so that fluctuating compressive forces acted on the pads. The range of movement was adjusted so that the minimum force was low, and the maximum force was similar in magnitude to the peak force expected to act on the paw pad during a 1-m jump down. Stresses and strains were recorded as outputs, and each test was allowed to run for at least 5 cycles, before a record was made. Finally, the magnitudes of energy dissipation and elasticity modulus were analyzed using an analysis of variance (ANOVA). An F-test was performed to determine the statistical significance of the test data at p of 0.05.

### 2.5. Finite Element Modelling

Due to the irregularity of the reconstructed compartment structure, we idealized it as an ellipsoid in this paper. Meanwhile, in combination with previous studies [10, 19], we also established a cylinder for control, using a commercially available software SolidWorks. The base radius of the cylindrical model is 20 mm and the height is 60 mm. The radius of the three axes of the ellipsoid are 20 mm, 20 mm and 45 mm, respectively. It is worth noting that, the ellipsoidal and cylindrical shell have an outward thickness of 1 mm to ensure that the two models have the same volume. The dimensions were designed to facilitate modeling and subsequent observation.

**Figure 2:**
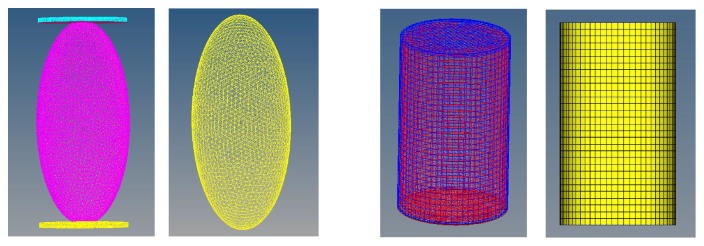
The ellipsoid and cylindrical models before and after mesh generation used for the finite element analysis.

In order to simulate the hydrodynamic effects, the shell models were filled with incompressible water. Furthermore, we applied the software Hypermesh 12.0 to mesh the models, as shown in Figure 2. The top and ground plates were placed at the upper and lower ends of the ellipsoid model, respectively, in order to facilitate the application of a loading force in the finite element calculation, and their materials were set as steel. Moreover, the material properties of water were indisputable, in line with the tradition. Based on a study [20] about finite element modelling of collagen fibers, the material properties of the shells were determined, and all material properties were provided in Table 1.

It can be seen from the previous cat jumping experiments, there was a nearly linear growth in the ground reaction force on the paw pads. In order to simulate the value of the peak force acting on the pad during a 1-m jump down, the dimensions of the finite element models were compared with the actual sizes of the adipose compartments, obtained from micro-CT scanning and section staining results, to derive the conversion relationship between them. And the actual peak force was then converted into a suitable force that was linearly loaded onto the two models (making the total force on the top surface increase to 20 N in 2.25s), in accordance with this conversion relationship. Therefore, in the simulation process, we used the load control instead of the displacement control. Finally, the effective stresses (Von Mises), displacements and shape change of the two models in the whole fluid-solid coupling analysis step were recorded and analyzed.

## 3. Results and Discussion

### 3.1. Macroscopic Mechanics

**Figure 3:**
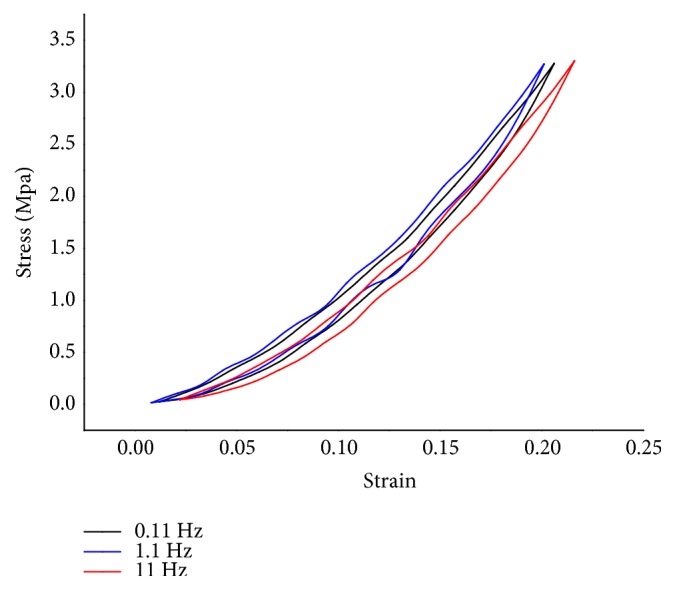
Representative average Stress-Strain curves of tests at 0.11Hz, 1.1Hz, and 11Hz.

The hysteresis loops of stress-strain at three frequencies are graphically presented in Figure 3, while associated energy dissipation and elasticity modulus at particular loads are provided in Table 2. Obviously, the pads have nonlinear, visco-elastic properties, in which the elastic modulus, defined as the gradient of the hysteretic loop, increases roughly as a pad is compressed. Thus, we suspect that the ground reaction force, however large, cannot reduce the pad thickness zero. It is logical to argue that the pads must not be too stiff for stability when the paws first touch the ground, and the subsequent gradual increase in stiffness can improve their compliance with the ground, thus preventing chattering. Additionally, the increased resistance to deformation can avoid injury to its paws due to the instantaneous large impact, for a given cat. Furthermore, the behaviour stiffness increases roughly in proportion to strain, appears similar to that of rubbery polymers [21], which are widely applied to vibration reduction and isolation, in turn, suggesting that the cat paw pads are indeed excellent shock absorbing materials.

As is shown in Table 2, the energy dissipations have values between 22 and 38 for all pads, in which no significant differences (*p* > 0.05) were found between all loading frequencies. Likewise, the elasticity moduluses at the two given loads do not show statistically significant difference (*p* > 0.05) for three frequencies. This property of cat paw pad seems to be comparable to that of human heel pad. In a study [18] on mechanical properties of human subcalcaneal fat pad in compression, the authors found that, although fluids (fat and water) were involved, its mechanical properties were only slightly affected by strain rate and temperature, suggesting that there was no bulk fluid flow between the small adipose compartments in the subcutaneous tissue. Notably, the comparison of results indicates that the septa appears to be arranged in a closed-cell type of structure, which is consistent with the findings of a previous study that it could not induce interseptal spread of India ink, even under significant pressure [22]. The adipose compartment of cats therefore can also be regarded as a model, which is filled with fluid and fixed volume, but shape deformation occurs during load-bearing.

### 3.2. Microstructure

**Figure 4:**
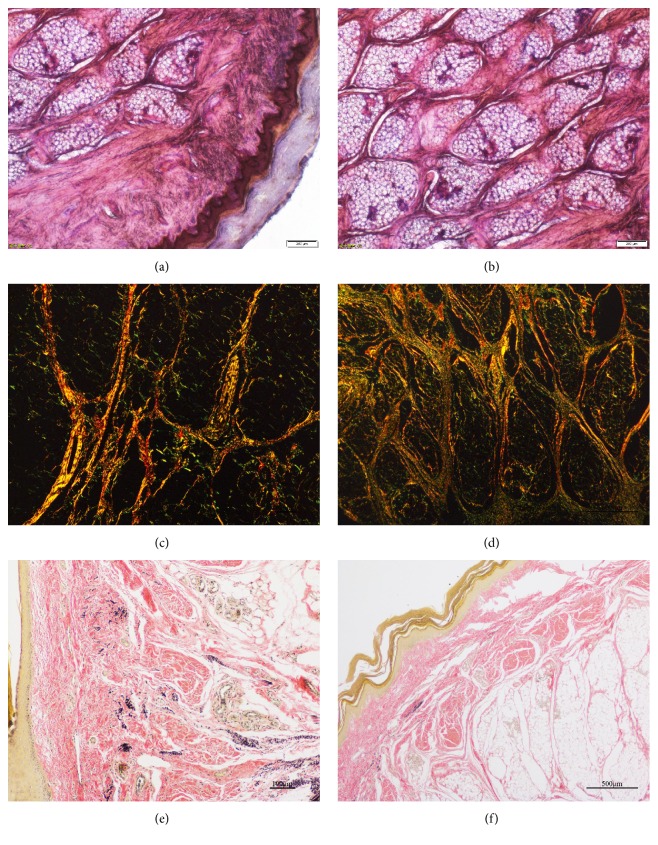
Representative histological images of the paw pad, stained with hematoxylin-eosin stain (a, b), Verhoeff-van Gieson stain (c, d), and Sirius red stain (e, f). Scale bars in the images (a–f) represent 200 *μ*m, 200 *μ*m, 100 *μ*m, 500 *μ*m, 100 *μ*m, and 500 *μ*m, respectively.

**Figure 5:**
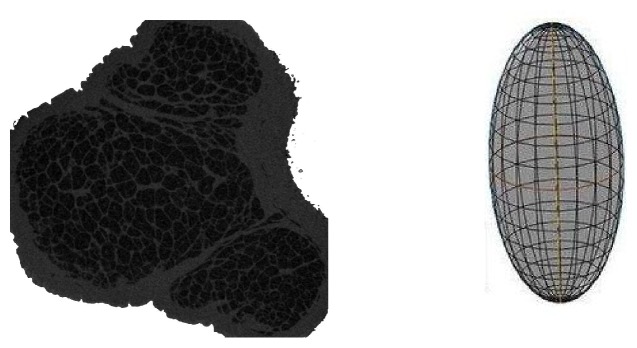
CT image (left) of the paw pad and idealized schematic diagram (right) of the spatial structure of an adipose compartment.

Histological examination and micro-CT scanning results of metacarpal pads from the left forelimbs are presented in Figures 4 and 5 and Table 3. It can be seen that the paw pads of cats have a typical layered structure, which can be roughly divided into three layers: the epidermis layer, the dermis layer and the subcutaneous layer. It is worth noting that in the dermis and subcutaneous layer, the fibers are mainly composed of collagen fibers, of which collagen type I is the majority. This is understandable, as type I collagen is mainly in the form of crude fibers, which have strong tensile strength and can bear high stress. In particular, in subcutaneous tissue, the collagenous membranes are consist mostly of collagen I fibers, and collagen III fibers are chiefly distributed inside the adipose compartments, surrounding and interspersing adipocytes. With regard to elastic fibers, they are mainly distributed in the dermis layer, while few elastic fibers is found in the subcutaneous layer. Since the epidermis layer that contacts the ground directly, is subjected to tremendous pad-ground wear, friction and impact during locomotion [23], it can be speculated that this layer is composed of the hardest material among the three layers. Besides, the dermis layer lies between the epidermis layer and the subcutaneous layer, in which the dermal papilla composed of matrix tissues is inserted into the epidermis layer [3], forming a honeycomb structure that helps absorb energy during impact [24, 25]. In the subcutaneous layer, it was found that there were many collagen fibers and fat cells, resulting in a large number of adipose compartments surrounded by collagenous membranes. Additionally, the subcutaneous layer is composed of the softest materials among the three layers, and it is the foremost energy absorber in the pad [10, 26]. Based on the results of mechanical testing, an adipose compartment can be regarded as a small hydrostatic system. Furthermore, similar layered structure are also found in the footpads of human beings, elephants, dogs and leopards [3, 7, 15, 26, 27].

However, thus far, little is known about the three-dimensional structure of adipose compartments in subcutaneous tissue. Therefore, we scanned the pads using micro-CT, in order to investigate the spatial structure of adipose compartments for the first time, and the results suggest that the three-dimensional structure of each compartment is not exactly the same, which can also be inferred from the results of section staining that, for a given section, each closed area has a different shape. However, they have one thing in common, that is, the cross-sectional area decreases gradually from the middle to the two ends, which is similar to the structure of the ellipsoid. In a departure from the previous study which reduced their structures to cylinders [10], it is suspected that this ellipsoid-like structure has more advantages in cushioning impact force. As a consequence, in the subsequent finite element study, we idealized the structure of an adipose compartment into an ellipsoid, and established a cylindrical model as a reference to investigate the micromechanical response of the compartment under impact force.

### 3.3. Finite Element Analysis

**Figure 6:**
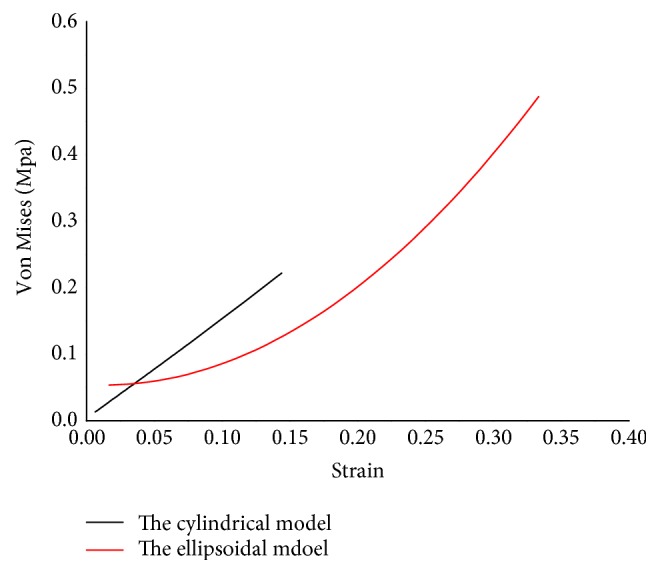
The stress (Von Mises) versus strain curves of the ellipsoidal and cylindrical models.

The comparison between ellipsoidal and cylindrical models using the stress (Von Mises) vs strain curves is shown in Figure 6, in which it can be seen that the maximum Von Mises and strain of the ellipsoid were obviously larger than those of the cylindrical. The result suggests that compared with the cylindrical, the ellipsoid model can absorb more shock energy by larger strain and stress. Additionally, it can be observed that the cylindrical structure is more resistant to deformation, which means it is more rigid. As mentioned in the previous study [13], a paw with pure elastic pads might be set into oscillation in the early stage of impact, resulting in the instant loss of contact with the ground, making the paw likely to shift its position. It is logical to argue that, at the beginning of the step, the smaller stiffness and larger deformation of the paw pad, which is exactly found in our ellipsoidal model, can avoid the occurrence of the above phenomenon, and thus maintain stability.

**Figure 7:**
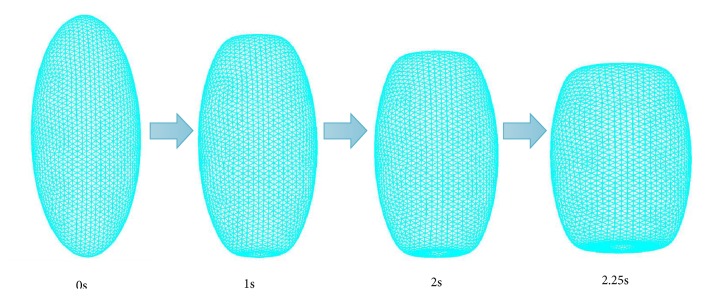
The shape change of the ellipsoidal model during loading.

Furthermore, the stress-strain curve of the cylindrical model is almost linear, while that of the ellipsoid has an increasing slope. We suspect that the reason for this result is the shape of the cylinder is always a cylinder during the compression process, only the height and width have changed, but the shape of the ellipsoid has changed a lot. Accordingly, we also analyzed the shape change of the ellipsoidal model during loading, as is shown in Figure 7, finding that the shape indeed gradually became cylindrical with increasing pressure. This confirms that the change in shape to a cylinder with greater resistance to deformation results in an increasing stiffness of the ellipsoid during loading. Interestingly, this characteristic the stiffness increases was also found in the results of mechanical tests on the paw pads. Therefore, it can be speculated that the shape change of the microscopic adipose compartments may be the reason why the macroscopic stiffness increased with increasing time after contact.

**Figure 8:**
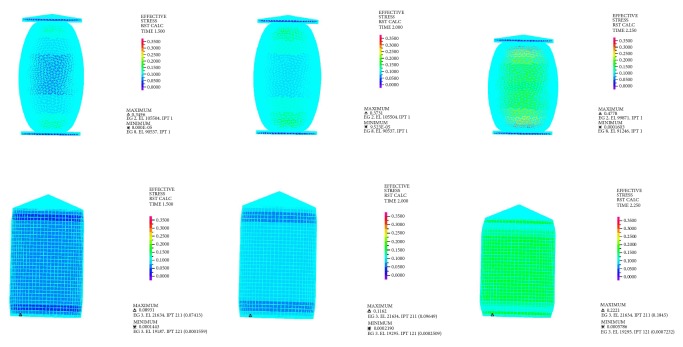
The color nephograms of the Von Mises of the two models during loading.

In order to further investigate the stress distribution characteristics, the color nephograms of the Von Mises of the two models at different loading times were provided in Figure 8. It was found that the Von Mises were much higher at both ends than in the middle of the ellipsoidal model, while, the stresses on the cylindrical model were nearly uniformly distributed. Besides, it can be inferred that for the ellipsoid model, there was a transmission of stress from both ends to the middle as the compressive force increased, in order to ensure that when its shape into a cylinder, the distribution of stresses can be close to uniform. Obviously, the stress distribution characteristic of the ellipsoid model determines that the deformation of both ends of the ellipsoid is relatively large at the initial impact stage, which makes its shape change to the cylindrical, further leading to an increase in the macroscopic stiffness. We also suspect that the behavior of stress transmission may be a protective mechanism of the paw pad during impact. There is no doubt that these features are combined to confer a better ability of resistance impact for the cat's paw pad.

## 4. Conclusions

By summarizing the results of mechanical testing, histological examination, micro-CT scanning and finite element analysis, we are able to examine the microstructure of the cat's paw pad and to analyze the macroscopic and microscopic mechanical responses to impacts, gaining insight into the biomechanism of impact resistance in cat's paw pad. Our results show that there are variable nonlinear and viscoelastic properties in cat's paw pads, which is attributable to the changes in the shapes of the microscopic adipose compartments during impact. Notably, the pads were found to have multiple layers, which help dissipate the impact forces, and the ellipsoid-like structures of adipose compartments exert a dominant role in impact resistance. The results of this study can provide biological inspiration for impact resistant foot pad to reduce human lower limbs injuries during landing. It should be noted that, in this study, we ignored the interplay between the adipose compartments, while, in fact, the space arrangement of compartments would affect the energy absorption and shape change of pads during impact, so further study is warranted to establish the three-dimensional structure of multiple compartments simultaneously.

## Figures and Tables

**Table 1:** Material properties of all components used for finite element modelling.

	Ellipsoid shell	Cylindrical shell	Water	Top and ground plates
Fluid Bulk Modulus (Mpa)	-	-	2000	-
Young's Modulus (Mpa)	1	1	-	21300
Poisson's Ratio	0.3	0.3	-	0.3
Density (t/mm^3^)	1.27e-009	1.27e-009	1e-009	1e-018
Viscosity (Mpa·s)	-	-	1e-009	-

**Table 2:** Means and SD of energy dissipation and elasticity modulus at particular loads, for different vibration frequencies.^a^

Frequency (Hz)	Elasticity modulus (Mpa) at loads of		Energy dissipation (%)^*∗*^
	50N^*∗*^	100N^*∗*^	
0.11	6.52 (1.38)	14.83 (3.14)	29.19 (4.13)
1.1	7.96 (2.56)	15.33 (2.83)	31.28 (5.54)
11	8.21 (2.87)	16.18 (1.65)	27.67 (3.87)

^a^Values in parentheses are the standard deviations (SD). ^*∗*^Parameter does not show statistically significant difference between loading frequencies.

**Table 3:** Composition of fibers in the dermis layer and subcutaneous layer investigated.

	Elastic fibers	Collagen I fibers	Collagen III fibers	Other components
Dermis layer	9%	53%	24%	14%
Subcutaneous layer	<1%	67%	22%	11%

## Data Availability

The data used to support the findings of this study are available from the corresponding author upon request.
